# Supplementary material for: Regional uniqueness of tree species composition and response to forest loss and climate change
Source: Nat Commun. 2024 May 31;15:4375. doi: 10.1038/s41467-024-48276-3 (PMC11143270; doi:10.1038/s41467-024-48276-3)
Supplement: Supplementary file 1 — Supplementary Information [file 41467_2024_48276_MOESM1_ESM.pdf]

Supplementary Figures for “Regional uniqueness of tree species composition and response to forest loss and climate change”

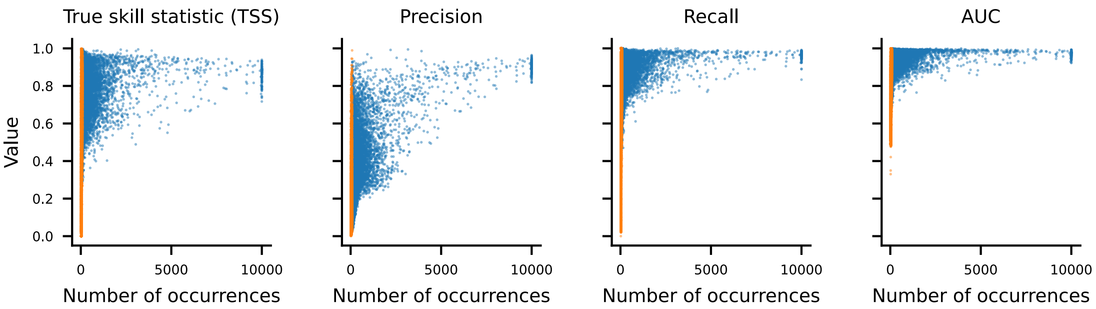

Supplementary Figure 1. **Model performance drops for species with very few occurrences.** Number of occurrences in training data against different model performance metrics, true skill statistic (TSS), precision, recall, and area under the ROC curve (AUC), computed on a 3-fold cross-validation for n=24,140 species, showing that low performance are obtained by species with less than 90 occurrences in the training data, shown here in orange.

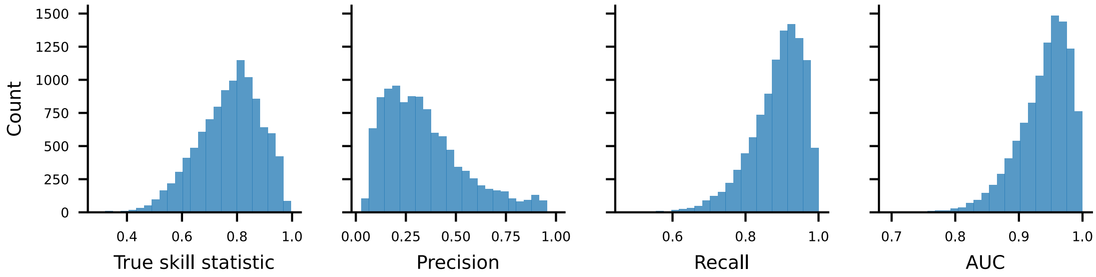

Supplementary Figure 2. **Distribution of cross-validated model performance.** Histogram of model performance metrics computed on 3-fold cross-validation for the n=10,590 species with at least 90 occurrences.

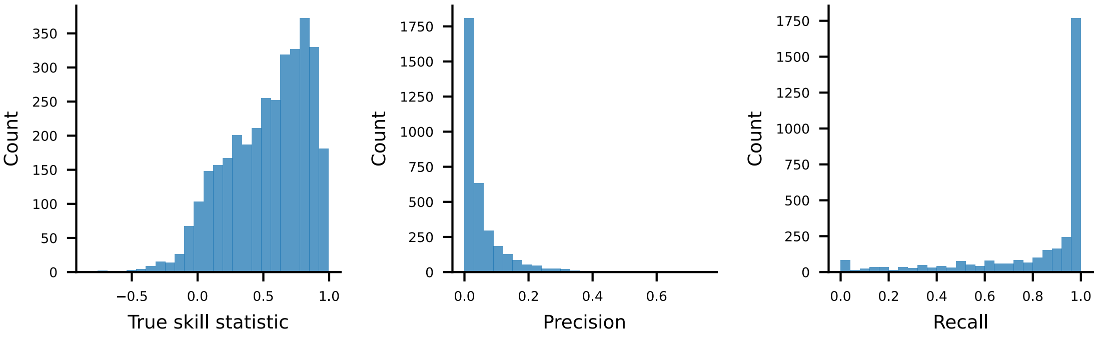

Supplementary Figure 3. **Distribution of model performance on independent data.** Histograms of validation metrics when assessing our models on an independent dataset, sPlot presence-absence data, for n=3,594 species for which at least 5 observations were recorded in sPlot in the area that was considered in our modeling approach. We included true skill statistic (TSS), precision, and recall.

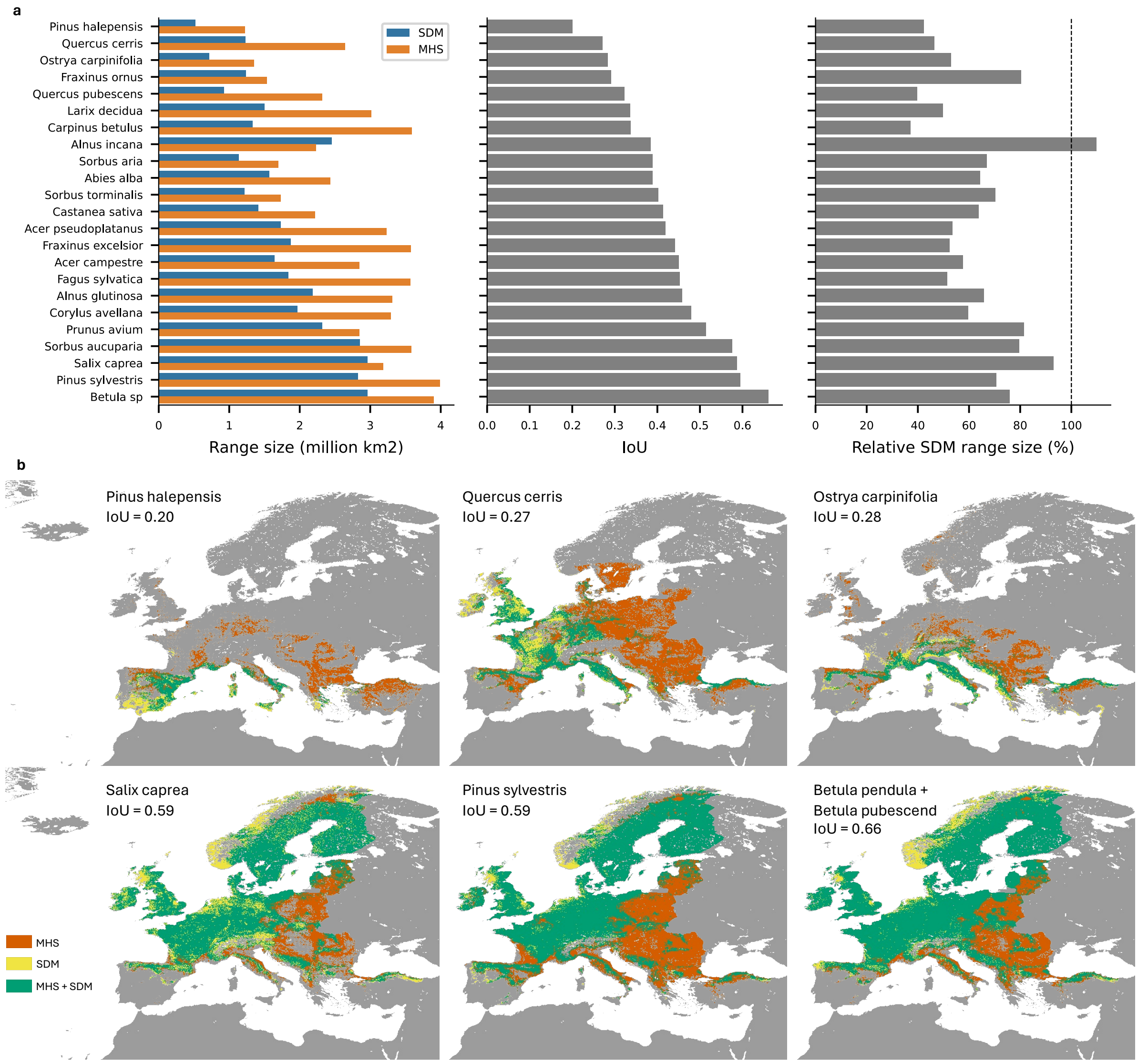

Supplementary Figure 4. **Comparison of the ranges of our modeled species' distributions (SDM) and maximum habitat suitability (MHS) maps for n=23 species for which data was available from the "Tree species distribution data and maps for Europe" report from the European Commission. a.** Bar plot of the range sizes of SDM and MHS, intersection over union (IoU) and relative SDM range size (SDM range size divided by MHS range size, line at 100% indicates equal range size) for each species. **b.** Maps of SDM and MHS predictions for n=3 species that obtained the lowest IoU values (top row) and n=3 species that obtained the highest IoU values (bottom row). Note that *Betula pendula* and *Betula pubescens* were combined in one MHS map. The maps were created with QGIS and the grey base map corresponds to all areas for which model predictors were available.

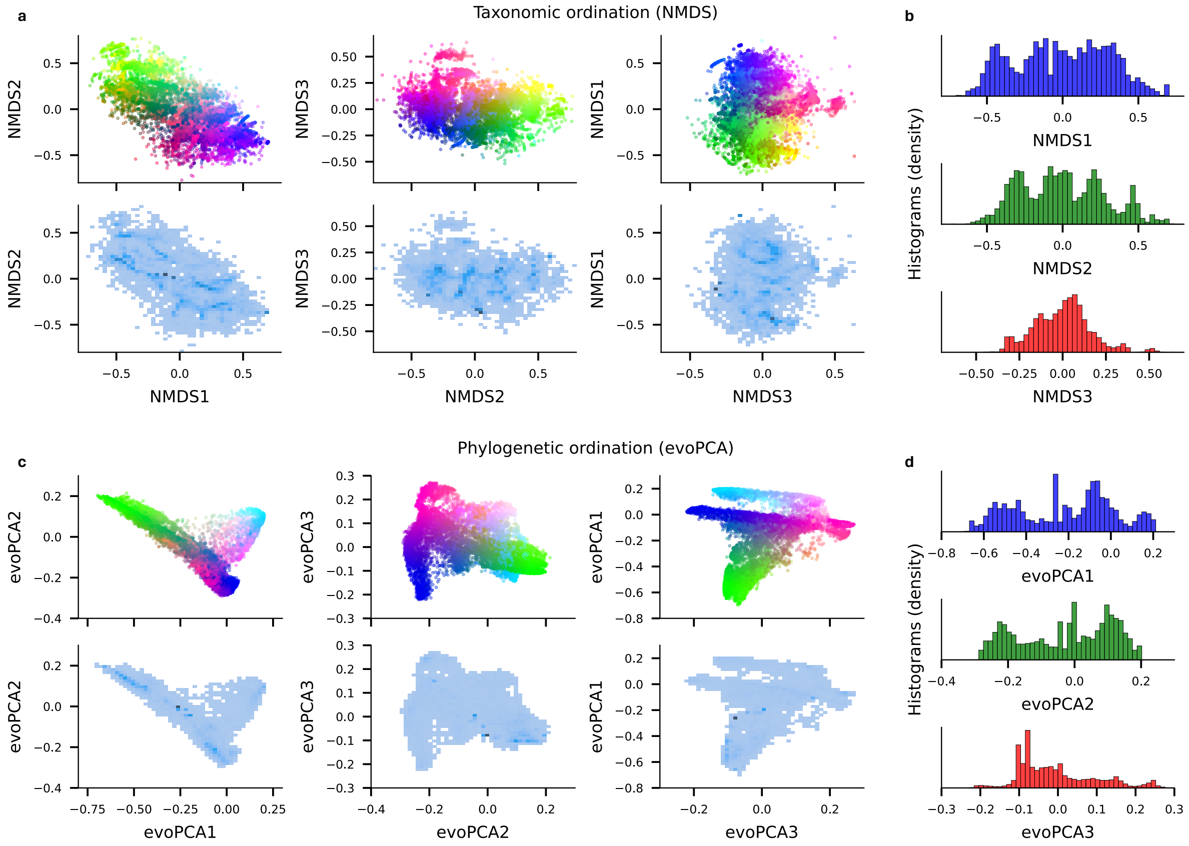

Supplementary Figure 5. **Additional views of the taxonomic (NMDS) and phylogenetic (evoPCA) composition ordinations shown in Figure 1.** These ordinations are computed on the global community matrix derived from the modeled distributions of n=10,590 tree species sampled at a resolution of 100 km, resulting in n=12,548 sites. **a,c.** Three-dimensional taxonomic and phylogenetic ordinations shown in two-dimensional scatter plots with colors mapping the position in the three-dimensional ordinations (top row), and shown in two-dimensional histograms, where darker colors indicate higher densities (bottom row). **b,d.** Histograms of each axis of the taxonomic and phylogenetic ordinations, plotted in the color used in the color mapping. Note that the 3 axes of each ordination are mapped to red, green, and blue with minimum and maximum values corresponding to the 10th and 90th percentiles.

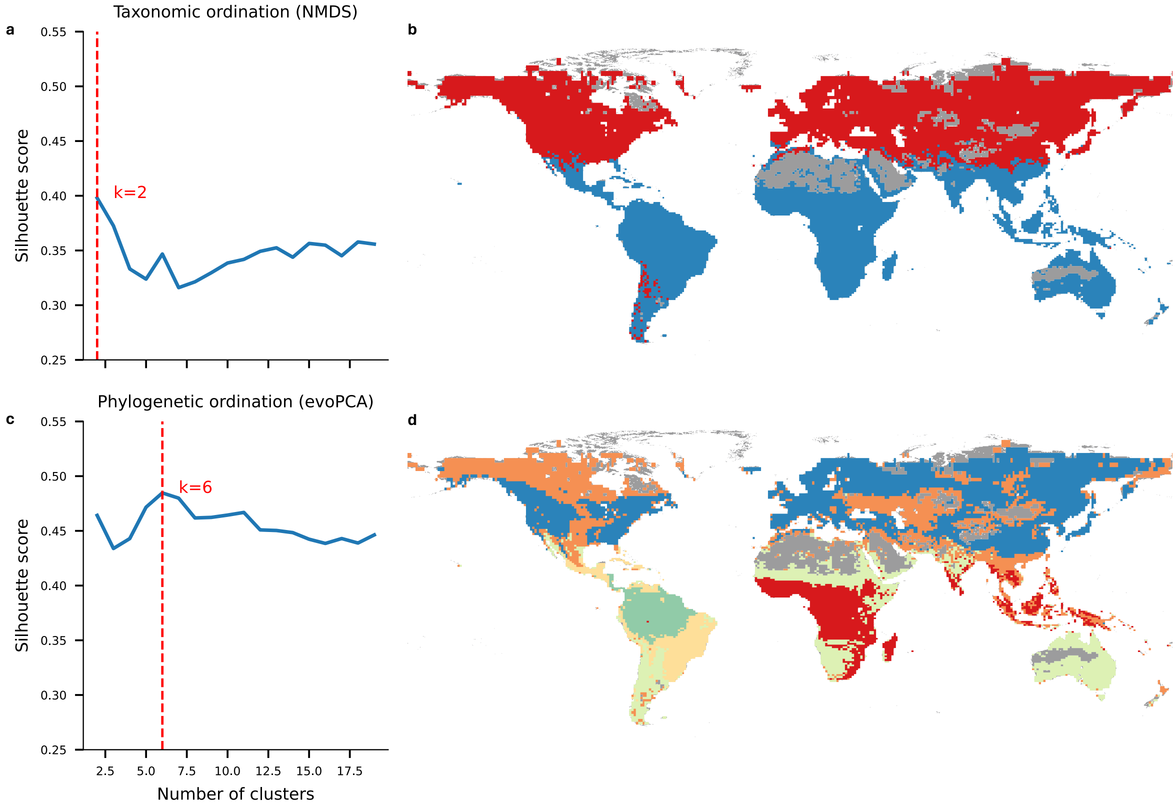

Supplementary Figure 6. **Clustering of taxonomic (NMDS) and phylogenetic (evoPCA) diversity maps generating two and six clusters, respectively, using the silhouette clustering score. a,c.** Plots of silhouette scores for increasing clusters using the k-means clustering algorithm for both taxonomic and phylogenetic ordinations. **b,d.** Global maps of clustered taxonomic and phylogenetic ordinations using the number of clusters yielding the best silhouette score. The maps were created with QGIS and the grey base map corresponds to all areas for which model predictors were available.

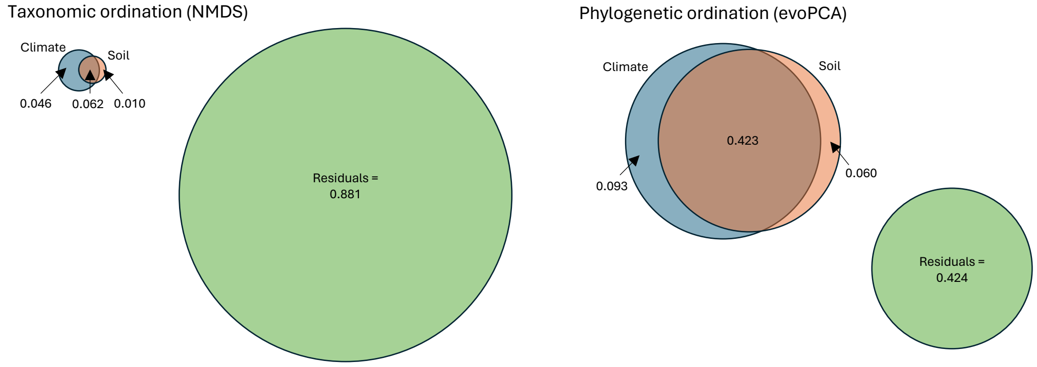

Supplementary Figure 7. **Variation partitioning analysis for taxonomic and phylogenetic ordinations.** The size of the circles and the numbers indicate variance explained by the climatic variables (mean annual temperature, temperature seasonality, annual precipitation, precipitation seasonality, growing season length, and potential net primary production), the soil variables (soil pH, coarse fragment content, and silt content), by both sets of variables simultaneously, and the residual variance not explained by either set of variables, in each ordination.

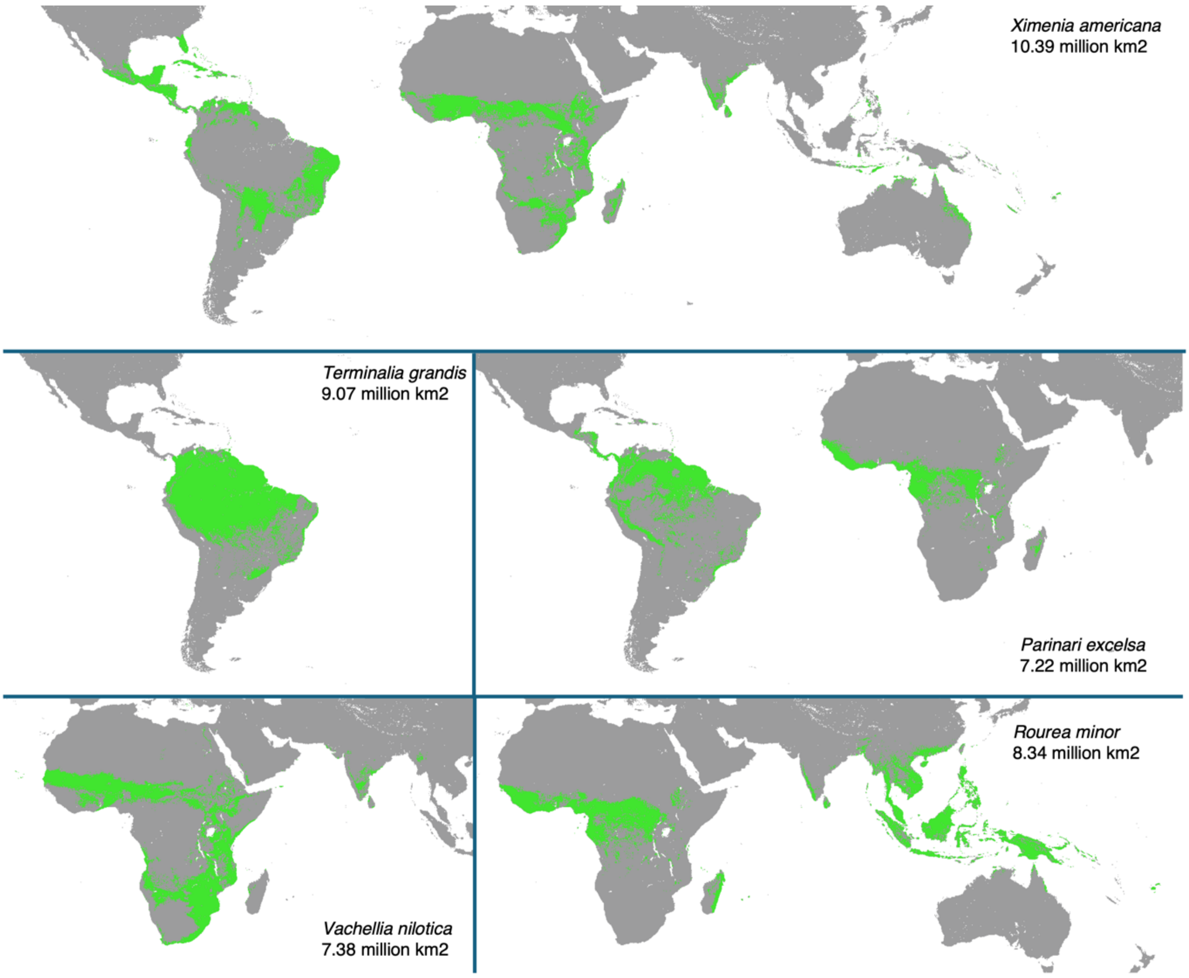

Supplementary Figure 8. **SDM output for tropical species with vast ranges predicted by our models.** The maps were created with QGIS and the grey base map corresponds to all areas for which model predictors were available.

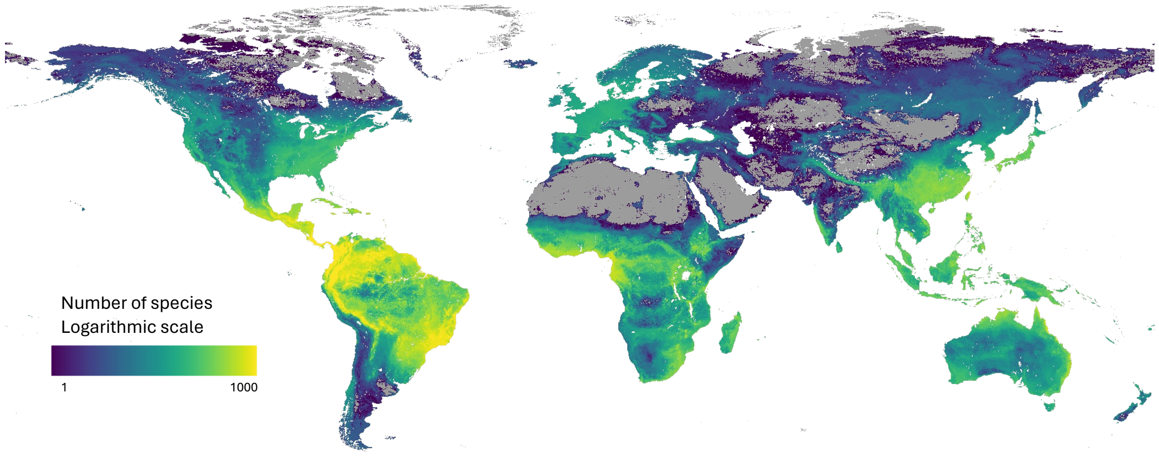

Supplementary Figure 9. **Global map of the number of species predicted by our SDMs at 30-arc second resolution.** This map reflects global patterns of tree species richness although it contains only the distributions of n=10,590 species. Missing species are most likely rare species, as well as species from areas that are less well sampled. The maps were created with QGIS and the grey base map corresponds to all areas for which model predictors were available.
