# Supplementary material for: Regional uniqueness of tree species composition and response to forest loss and climate change
Source: Nat Commun. 2024 May 31;15:4375. doi: 10.1038/s41467-024-48276-3 (PMC11143270; doi:10.1038/s41467-024-48276-3)
Supplement: Supplementary file 3 — Reporting Summary [file 41467_2024_48276_MOESM3_ESM.pdf]

Corresponding author(s): Nina van Tiel

Last updated by author(s): Apr 15, 2024

Reporting Summary

Nature Portfolio wishes to improve the reproducibility of the work that we publish. This form provides structure for consistency and transparency in reporting. For further information on Nature Portfolio policies, see our Editorial Policies and the Editorial Policy Checklist.

Statistics

For all statistical analyses, confirm that the following items are present in the figure legend, table legend, main text, or Methods section.

n/a	Confirmed	
☐	☒	The exact sample size (*n*) for each experimental group/condition, given as a discrete number and unit of measurement
☒	☐	A statement on whether measurements were taken from distinct samples or whether the same sample was measured repeatedly
☐	☒	The statistical test(s) used AND whether they are one- or two-sided<br>*Only common tests should be described solely by name; describe more complex techniques in the Methods section.*
☐	☒	A description of all covariates tested
☒	☐	A description of any assumptions or corrections, such as tests of normality and adjustment for multiple comparisons
☐	☒	A full description of the statistical parameters including central tendency (e.g. means) or other basic estimates (e.g. regression coefficient) AND variation (e.g. standard deviation) or associated estimates of uncertainty (e.g. confidence intervals)
☐	☒	For null hypothesis testing, the test statistic (e.g. F, t, r) with confidence intervals, effect sizes, degrees of freedom and P value noted<br>*Give P values as exact values whenever suitable.*
☒	☐	For Bayesian analysis, information on the choice of priors and Markov chain Monte Carlo settings
☒	☐	For hierarchical and complex designs, identification of the appropriate level for tests and full reporting of outcomes
☒	☐	Estimates of effect sizes (e.g. Cohen's d, Pearson's r), indicating how they were calculated

Our web collection on statistics for biologists contains articles on many of the points above.

Software and code

Policy information about availability of computer code

Data collection	All data was downloaded from online databases. Data pre-processing was done with R, python and Google Earth Engine.
Data analysis	All data analysis was done with R, python and Google Earth Engine. All code necessary to repeat the study is available in this GitHub repository: https://github.com/ninavantiel/tree_sdms (https://zenodo.org/doi/10.5281/zenodo.10908653).<br>Python v 3.8.13 was used with the following packages: pandas v1.4.4, numpy v1.23.4, scikit-learn v1.1.3, earthengine-api v0.1.329, matplotlib v3.5.3, seaborn v0.12.1.<br>R v4.2.2 was used with the following packages: tidyr 1.3.0, tidyverse 1.3.2, tibble 3.1.8, data.table 1.14.6, dplyr 1.1.0, gridExtra 2.3, V.PhyloMaker 0.1.0, adiv 2.2, vegan 2.6.4, ggplot2 3.4.1

For manuscripts utilizing custom algorithms or software that are central to the research but not yet described in published literature, software must be made available to editors and reviewers. We strongly encourage code deposition in a community repository (e.g. GitHub). See the Nature Portfolio guidelines for submitting code & software for further information.

Data

Policy information about availability of data

All manuscripts must include a data availability statement. This statement should provide the following information, where applicable:

- Accession codes, unique identifiers, or web links for publicly available datasets
- A description of any restrictions on data availability
- For clinical datasets or third party data, please ensure that the statement adheres to our policy

The occurrence data used in this study are available from the online databases:
- Botanical Information and Ecology Network (BIEN): https://bien.nceas.ucsb.edu/
- BIOMASS: https://www.nature.com/articles/sdata201770#data-records-
- Caudullo et al. 2017: https://doi.org/10.1016/j.dib.2017.05.007
- CONIFER:https://herbaria.plants.ox.ac.uk/bol/conifers
- DRYFLOR: http://www.dryflor.info/data/datadownload108
- GBIF: https://www.gbif.org/occurrence/download/0032444-200221144449610
- GFBI: https://www.gfbinitiative.org/
- IDIGBIO: https://www.idigbio.org/portal/search
- INDIABIODIVERSITY: https://indiabiodiversity.org/observation/list
- PNG: http://www.pngplants.org/search.htm
- PREDICTS: https://data.nhm.ac.uk/dataset/the-2016-release-of-the-predicts-database
- RAINBIO: https://gdauby.github.io/rainbio/download_page.html

Additionally, one unpublished dataset (MUSEUM) was used; it is available from the corresponding author upon request.

The bioclimatic raster data used as used model covariates in this study are available from CHELSA 2.1 (https://chelsa-climate.org/downloads/).
The edaphic raster data used as used model covariates in this study are available from Soilgrids (https://www.isric.org/explore/soilgrids).
The reported native ranges data used in this study are available from GlobalTreeSearch (https://tools.bgci.org/global_tree_search.php).
The country boundary data from the FAO used in this study are available through the Google Earthengine data catalogue (https://developers.google.com/earth-engine/datasets/catalog/FAO_GAUL_2015_level0).
Plot data used as an independent validation data in this study area available from sPlot (https://www.idiv.de/en/splot.html).
The biome and ecoregion database used in this study are available through the Google Earthengine data catalogue (https://developers.google.com/earth-engine/datasets/catalog/RESOLVE_ECOREGIONS_2017).
The tree cover map used in this study are available through the Google Earthengine data catalogue (https://developers.google.com/earth-engine/datasets/catalog/UMD_hansen_global_forest_change_2023_v1_11).

Source Data for Figures 1, 2 and 3 are provided with this paper as Source Data files.
The raster data of the modelled tree species' distributions generated in this study have been deposited in Zenodo (https://doi.org/10.5281/zenodo.10911892).

Research involving human participants, their data, or biological material

Policy information about studies with human participants or human data. See also policy information about sex, gender (identity/presentation), and sexual orientation and race, ethnicity and racism.

Reporting on sex and gender	N/A
Reporting on race, ethnicity, or other socially relevant groupings	N/A
Population characteristics	N/A
Recruitment	N/A
Ethics oversight	N/A

Note that full information on the approval of the study protocol must also be provided in the manuscript.

Field-specific reporting

Please select the one below that is the best fit for your research. If you are not sure, read the appropriate sections before making your selection.

☐ Life sciences ☐ Behavioural & social sciences ☒ Ecological, evolutionary & environmental sciences

For a reference copy of the document with all sections, see nature.com/documents/nr-reporting-summary-flat.pdf

Ecological, evolutionary & environmental sciences study design

All studies must disclose on these points even when the disclosure is negative.

Study description	We estimated the spatial distribution of a large number of tree species at the global level. We used a cloud-based implementation of an environmental niche model algorithm with geographic dispersal constraints and over 26 million recorded observations of trees from a broad compilation of databases to generate ranges for 10,590 tree species for which we had sufficient occurrence data. Subsequently, and beyond the pure large-scale mapping effort, we used the obtained distributions to perform three analyses investigating the global distribution of tree species which are presented in the manuscript.
Research sample	The study was conducted on all tree species for which we were able to collect enough data. We used the checklist from GlobalTreeSearch from BGCI to define which species to consider as trees. Occurrence data was downloaded from several databases. Links to each of these databases can be found in the Methods and Data Availability section.
Sampling strategy	We considered tree species for which we were able to obtain 20 distinct observations. After we fit our models and evaluated them, we decided to consider only the results of models that were fit on at least 90 distinct observations.
Data collection	Data was downloaded from the Internet from the different databases cited in the Methods and Data Availability sections. The data collection work took place between 2019 and 2020. No field data collection was performed for this study.
Timing and spatial scale	The timing of the occurrence data collected was not considered. We considered data for species globally.
Data exclusions	We excluded data points for which we did not have model covariate values, typically observations that fell outside of the covariate pixel grid because they were too close to the coast. We also merged observations of the same species that fell in the same gridcell of the pixel grid we used for modelling. Finally, we excluded species' observations that fell outside of a polygon constructed based on each species' reported native range (obtained from BGCI's GlobalTreeSearch).
Reproducibility	We did not take concrete measures for the reproducibility of the occurrence dataset. However, we expect these database to only include more data in the future, therefore we expect the data we used to still be available for future research. The code to reproduce our workflow is available in a GitHub repository linked in the paper.
Randomization	We sampled the data points uniformly at random for each species for model validation with k-fold cross validation. Otherwise, no group allocation or randomization was necessary.
Blinding	No blinding was performed for our data analysis.

Did the study involve field work? ☐ Yes ☒ No

Reporting for specific materials, systems and methods

We require information from authors about some types of materials, experimental systems and methods used in many studies. Here, indicate whether each material, system or method listed is relevant to your study. If you are not sure if a list item applies to your research, read the appropriate section before selecting a response.

Materials & experimental systems

n/a	Involved in the study
☒	☐ Antibodies
☒	☐ Eukaryotic cell lines
☒	☐ Palaeontology and archaeology
☒	☐ Animals and other organisms
☒	☐ Clinical data
☒	☐ Dual use research of concern
☒	☐ Plants

Methods

n/a	Involved in the study
☒	☐ ChIP-seq
☒	☐ Flow cytometry
☒	☐ MRI-based neuroimaging

Plants

Seed stocks	N/A
Novel plant genotypes	N/A
Authentication	N/A
